# Is Peripheral Motion Detection Affected by Myopia?

**DOI:** 10.3389/fnins.2021.683153

**Published:** 2021-06-07

**Authors:** Junhan Wei, Deying Kong, Xi Yu, Lili Wei, Yue Xiong, Adeline Yang, Björn Drobe, Jinhua Bao, Jiawei Zhou, Yi Gao, Zhifen He

**Affiliations:** ^1^State Key Laboratory of Ophthalmology, Optometry and Vision Science, School of Ophthalmology and Optometry, Affiliated Eye Hospital, Wenzhou Medical University, Wenzhou, China; ^2^WEIRC, WMU-Essilor International Research Centre, Wenzhou, China; ^3^R&D AMERA, Essilor International, Singapore, Singapore

**Keywords:** myopia, peripheral vision, motion perception, motion speed, eccentricity

## Abstract

**Purpose:**

The current study was to investigate whether myopia affected peripheral motion detection and whether the potential effect interacted with spatial frequency, motion speed, or eccentricity.

**Methods:**

Seventeen young adults aged 22–26 years participated in the study. They were six low to medium myopes [spherical equivalent refractions −1.0 to −5.0 D (diopter)], five high myopes (<-5.5 D) and six emmetropes (+0.5 to −0.5 D). All myopes were corrected by self-prepared, habitual soft contact lenses. A four-alternative forced-choice task in which the subject was to determine the location of the phase-shifting Gabor from the four quadrants (superior, inferior, nasal, and temporal) of the visual field, was employed. The experiment was blocked by eccentricity (20° and 27°), spatial frequency (0.6, 1.2, 2.4, and 4.0 cycles per degree (c/d) for 20° eccentricity, and 0.6, 1.2, 2.0, and 3.2 c/d for 27° eccentricity), as well as the motion speed [2 and 6 degree per second (d/s)].

**Results:**

Mixed-model analysis of variances showed no significant difference in the thresholds of peripheral motion detection between three refractive groups at either 20° (*F*[2,14] = 0.145, *p* = 0.866) or 27° (*F*[2,14] = 0.475, *p* = 0.632). At 20°, lower motion detection thresholds were associated with higher myopia (*p* < 0.05) mostly for low spatial frequency and high-speed targets in the nasal and superior quadrants, and for high spatial frequency and high-speed targets in the temporal quadrant in myopic viewers. Whereas at 27°, no significant correlation was found between the spherical equivalent and the peripheral motion detection threshold under all conditions (all *p* > 0.1). Spatial frequency, speed, and quadrant of the visual field all showed significant effect on the peripheral motion detection threshold.

**Conclusion:**

There was no significant difference between the three refractive groups in peripheral motion detection. However, lower motion detection thresholds were associated with higher myopia, mostly for low spatial frequency targets, at 20° in myopic viewers.

## Introduction

The human visual system is not fully functional at birth. During early postnatal development, the eye grows toward emmetropia (Banks, 1980; Mohindra and Held, 1980). Abnormal development of the visual system can lead to different types of eye disorders, the most widespread of which is myopia (Wiesel and Raviola, 1977; Wallman et al., 1978; Siegwart and Norton, 2011). Myopia is a benign disorder in which visual images come to a focus in front of the retina mostly due to the elongation of the eye horizontally. The condition is mainly manifested as a reduction of distance visual acuity (Morgan et al., 2012). The global prevalence of myopia has reached very high as reported in multiple epidemiological studies (Grosvenor and Scott, 1993, 1994; Dirani et al., 2009; Lim et al., 2012), particularly in Asian populations (Wang et al., 2020; Xie et al., 2020). It was predicted that almost half of the world population would have myopia by 2050 (Holden et al., 2016).

Besides poor visual acuity, myopes also show abnormalities in spatial visual processing including contrast sensitivity (Stoimenova, 2007; Ehsaei et al., 2013), blur perception (Gwiazda et al., 1993; Rosenfield and Abraham-Cohen, 1999; Vasudevan et al., 2006; Maiello et al., 2017; Ang et al., 2020), color vision (Garcia-Domene et al., 2018), binocular vision (Vera-Diaz et al., 2018), and attention (Kerber et al., 2016). Myopes may also have abnormal temporal visual processing abilities. For example, Vera-Diaz et al. (2018) found that myopes had poorer performance than emmetropes in perceiving flickered binocular stimuli at lower temporal frequencies. Another study found that critical flicker frequency was lower in high myopes compared to emmetropes; and in a large range (5–60 Hz) of temporal frequency, the contrast modulation threshold of flickering stimuli was higher in high myopes (Chen et al., 2000). A recent study using a psychophysical multichannel functional test (Antón et al., 2012) to study the sensitivity of visual pathways in high myopes (Garcia-Domene et al., 2018) found that the sensitivity of the magnocellular pathway, which is mainly responsible for the motion perception (Merigan and Maunsell, 1990), decreased. Therefore, it suggested myopia may have impaired motion perception.

Most of the aforementioned studies focused on the central vision. Recent studies have shown that myopia also causes abnormal changes in the morphology of the peripheral retina (Ohno-Matsui et al., 2016; Nagra et al., 2018), and peripheral defocus is closely related to the development of myopia (Smith et al., 2005, 2010). In general, peripheral vision refers to the area outside 2° eccentricity of the fovea and parafovea (Strasburger et al., 2011). Many myopia progression control lenses have been designed based on the finding of myopic peripheral defocus slowing down the elongation of the eyeball (Sankaridurg et al., 2019; Tarutta et al., 2019; Kaphle et al., 2020; Lam et al., 2020). Studying the characteristics of visual information processing in the periphery of myopic vision is thus of great significance to our understanding of myopia.

To understand temporal information processing in the peripheral visual field of myopes, peripheral motion detection is a good starting point. Peripheral motion perception, as a fundamental visual function of humans, affects a range of higher-level cognitive functions, including orienting, balance, visually guided action, and mobility (Marron and Bailey, 1982; Nakayama, 1985; Geruschat et al., 1998; Marigold, 2008), and closely relates to daily activities (Henderson et al., 2013). Previous studies on the effect of myopia on peripheral motion perception did not reach consistent conclusions. To illustrate, Leibowitz et al. (1972) and Johnson and Leibowitz (1974) found no significant difference between myopes and emmetropes in motion discrimination task at 10°–80° eccentricities in the temporal visual field. McKee and Nakayama (1984) found that correcting peripheral refractive errors in myopes did not improve the performance of differential motion perception tasks in the lower peripheral visual field. A recent study (Kuo et al., 2018) assessed central and peripheral motion perception (at 3.65° and 12° eccentricities) using the random-dot paradigm also did not find significant differences in peripheral motion perception tasks including minimum displacement (Dmin), maximum displacement (Dmax), and motion coherence tasks between young myopic and emmetropic adults. However, they have found a small but significant correlation between the peripheral Dmin threshold in the superior-temporal visual field and the axial length, as well as the macular thickness of the corresponding inferior-nasal retina. The latter finding suggested that peripheral motion perception might be affected by myopia.

These studies have enriched our knowledge of the link between myopia and peripheral motion perception. However, considering that motion perception is influenced by a variety of factors, such as eccentricity (Leibowitz et al., 1972; Rogers, 1972; Johnson and Leibowitz, 1974; Koenderink et al., 1978a, b; McKee and Nakayama, 1984; van de Grind et al., 1987; Wesemann and Norcia, 1992), spatial frequency (Koenderink et al., 1978a, b; van de Grind et al., 1987; Boulton and Baker, 1991; Beard et al., 1997; Bex and Dakin, 2003; Lappin et al., 2009), speed (Lagae et al., 1993; Lappin et al., 2009; Ananyev et al., 2019), and the area in the different visual fields (Kuo et al., 2018), it remains unclear that whether myopia affects peripheral motion processing and if so, whether the potential effect varied with these factors. In this study, we directly addressed this issue by measuring the motion detection thresholds of gratings in a four-alternative forced-choice task in blocks of eccentricity, spatial frequency, and speed in young adults with low to high myopia and emmetropes.

## Materials and Methods

### Participants

Six adults [mean age: 25.17 ± 0.37 years old; mean ± standard deviation (SD)] with low to medium myopia [LM group; spherical equivalent refraction (SER) between −1.0 and −5.0 D (diopter)], five adults (mean age: 25.4 ± 0.8 years old) with high myopia (HM group; SER less than −5.5 D) and six controls (EM group; mean age: 23.83 ± 1.07 years old) with SER between +0.5 and −0.5 D, participated in the current study. Refraction was done for each subject at the beginning of the study. Subjects were then grouped according to the refraction (Flitcroft et al., 2019). All myopes wore self-prepared, habitual soft contact lenses during the experimental session. Subjects’ best-corrected visual acuity was equal to or better than log MAR 0.0. All subjects had no history of ocular surgery, or other eye diseases. Observer’s dominant eye, which was determined using the card-in-the-hole test (Dane and Dane, 2004), was tested in this study. Details of the dominant eyes of participants are provided in Table 1.

**TABLE 1:** Clinical details of the participants.

	**Emmetropes (EM)**	**Low to medium myopes (LM)**	**High myopes (HM)**
*n*	6	6	5
Age (y)	23.83 ± 1.07	25.17 ± 0.37	25.4 ± 0.8
Gender (female/male) (*n*)	4/2	4/2	5/0
Best-corrected visual acuity (logMAR)	−0.11 ± 0.11	−0.08 ± 0.09	−0.06 ± 0.06
Refraction (D)	−0.04 ± 0.31	−3.13 ± 1.19	−6.03 ± 0.75
Refraction of contact lenses (DE)	–	−2.96 ± 1.14	−5.65 ± 0.8

*Data are mean ± SD.*

*DE, dominant eye; the logMAR VA of LM and HM were measured with the soft contact lens correction.*

This study adhered to the Declaration of Helsinki. Informed consent was obtained from all subjects after explaining the nature and possible consequences of the study. The study was approved by the Ethics Committee of the affiliated eye hospital of Wenzhou Medical University.

### Apparatus

Stimuli were generated and controlled by a PC running Matlab R2016b (MathWorks, Inc., Natick, MA, United States) with Psychtoolbox 3.0.14 (Brainard, 1997; Pelli, 1997; Kleiner et al., 2007). The stimuli were presented on a gamma-corrected ASUS PG278QR LED screen (ASUS Corp., China) with a 2,560 × 1,440 resolution and a 60-Hz refresh rate. The average background luminance was 37.5 cd/on the screen. During the measurement, observers viewed the screen monocularly with their dominant eye at a viewing distance of 27 cm. The untested eye was covered with an opaque patch. The whole experiment was carried out in a dark room to ensure the only light source was the display.

### Stimuli

**FIGURE 1:**
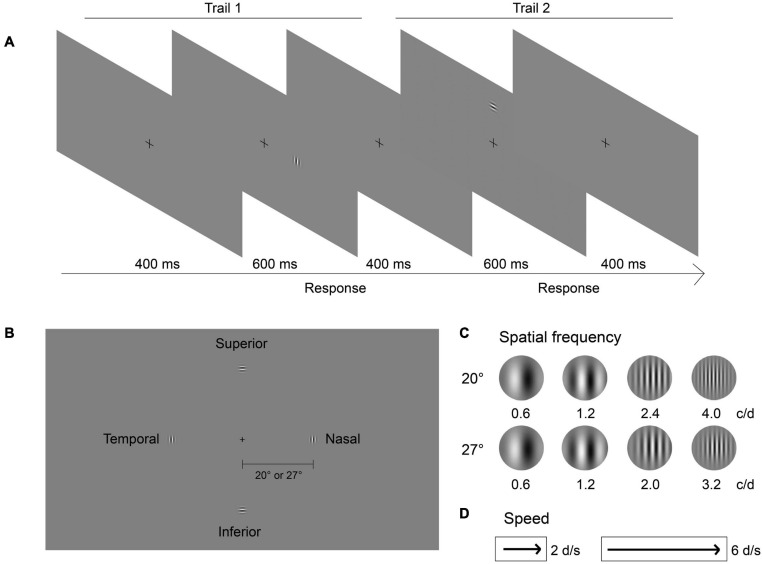
Illustration of the experiment design. **(A)** The peripheral motion detection procedure. In each trail, the stimulus was randomly presented in one of the four quadrants of the visual field for 600 ms. Then subjects were asked to determine in which quadrant the grating was presented, followed by a 400 ms interval before the onset of the next stimulus. **(B)** Examples of four quadrants (superior, inferior, nasal, and temporal) of the visual field. The cross in the center of the screen was for fixation. **(C)** Examples of SFs for two eccentricities, SF were 0.6, 1.2, 2.4, and 4.0 cycle/degree (c/d) at 20° eccentricity, and 0.6, 1.2, 2.0, and 3.2 c/d at 27° eccentricity. **(D)** Two speeds of grating moving were tested, 2 and 6 degree/second (d/s).

As shown in Figure 1, the target stimulus was a Gabor, which was a phase-shifting grating within a two-dimensional Gaussian window (sigma: 1.2° of visual angle; diameter: 4° of visual angle). The grating moved from the far periphery inward in the Gaussian window. The stimulus was presented randomly in one of the four quadrants of the visual field (Figure 1B), namely nasal, temporal, superior and inferior, at one of the two eccentricities (20° and 27°) on a uniform gray background. To be consistent with the major motion direction of environmental objects during locomotion, the motion direction of the target in each quadrant was aligned with its meridian. To avoid location or eccentricity change of the target, phase-shifting Gabors whose orientations were perpendicular to their motion directions were employed. Therefore, the orientation of the grating was vertical if the nasal and temporal visual fields were tested, or horizontal if the superior and inferior visual fields were tested. The spatial frequencies (SF) of the stimuli were 0.6, 1.2, 2.4, and 4.0 cycle per degree (c/d) at 20° eccentricity, and 0.6, 1.2, 2.0, and 3.2 c/d at 27° eccentricity (Figure 1C). Two speeds, 2 and 6 degrees per second (d/s) (Figure 1D), were tested.

### Procedure

All subjects underwent dark adaptation for 5 min in the darkroom before the measurements. Subjects were asked to look straight ahead, with their dominant eye, at a fixation cross in the center of the screen while performing the task. The non-dominant eye was covered with an opaque patch. A chin rest was used to minimize head movements to ensure the viewing distance and the eccentricities of the stimuli were corrected. The experiment was done in blocks of two eccentricities, four SFs, and two speeds with two repetitions in each combined condition, i.e., in total 32 runs. In each run, a four-alternative forced-choice task was employed.

Figure 1A shows the procedure of two trials. In each trial, a stimulus was presented randomly in one of the four quadrants of the visual field for 600 ms. We asked subjects to determine in which quadrant the target was presented, and to respond by pressing the “2,” “4,” “6,” and “8” keys on the keyboard, respectively. There was a constant 400 ms interval between the button press and the onset of the next stimulus. Two 2-down 1-up staircases were interleaved for each run to determine the contrast level of the gratings (Cornsweet, 1962). The step size of the staircase was 1 dB [Decibel, dB = 20^∗^log_10_ (C), C represents contrast (%)]. The contrast was started from the highest (100%), decreased with two consecutive, correct responses, and increased by one level with a single incorrect response. A reversal was defined as a change of direction of the staircases between increasing and decreasing. Each block was terminated after 50 trials or 10 reversals. The detection threshold was calculated as the mean level of the last five reversals of all four staircases under each condition. From the pilot testing, we found that the levels of contrast that were requested during the staircases at any spatial frequency, speed, and eccentricity in the current study did not exceed the resolution of the eight-bit graphic card’s capacity. Therefore, no special procedure to achieve extra bits was carried out.

One 5-min practice run was applied before the start of the test. Each run lasted about 5 mins. Subjects normally finished the test (which took 3.5–4 h in total) on two or three separate days within 1 week. Measures were taken during each session to prevent visual fatigue, including 2-min mandatory breaks after every one or two runs, and applying hydrating eye drops (Bausch & Lomb Incorporated) prepared by the authors for each subject who wore contact lenses.

### Statistical Analysis

Contrast thresholds of motion detection were used for statistical analysis. Two mixed-model analysis of variances (ANOVAs) were used to test the effects of one between-subjects factor-refractive group, and three within-subject factors-spatial frequency, speed, and quadrant of the visual field for each eccentricity. By Pearson correlation coefficient (ranging between −1 and 1; two-tailed) and empirical *p* values from permutation tests (based on 10,000 permutations of the data), which have been used to calculate the *p* values for multiple comparison correction, the correlation between the average motion detection threshold and the spherical equivalent were calculated. Statistical analysis was performed using Matlab and IBM-SPSS 23.0 (IBM Inc., Armonk, NY, United States).

## Results

### The Effect of Myopia on the Peripheral Motion Detection Threshold at 20° Eccentricity

**FIGURE 2:**
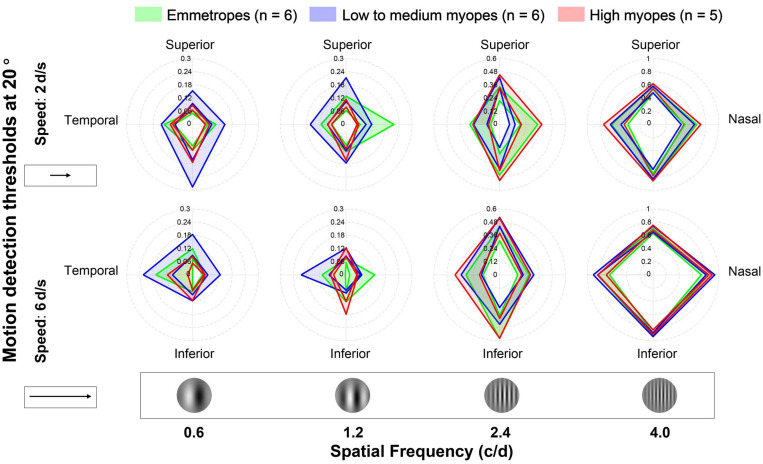
Average motion detection thresholds at 20° for emmetropes, low to medium myopes and high myopes. Data were plotted for different visual fields (in different quadrants), spatial frequencies (in different columns), and speeds (in different rows). The green, blue, and red lines and shadows represent three refractive groups (EM, LM, and HM). The width of the band represents the range between “mean ± standard error [SE]”.

In Figure 2, we plotted the average motion detection thresholds at 20° across visual fields, speeds, SFs for EM (green), LM (blue), and HM (red). LM group had larger peripheral motion detection thresholds than other groups at 0.6 c/d. At 1.2 c/d, the threshold of the nasal quadrant was higher in the EM group, and the threshold of temporal and superior quadrants at low speed were higher in the LM group. While at high SFs (i.e., 2.4 and 4.0 c/d), there was no obvious difference in the thresholds among the three groups. We conducted a four-factor, mixed model ANOVA with one between-subject factor (refractive group) and three within-subject factors (quadrant, SF, and speed), on peripheral motion detection thresholds. However, the ANOVA revealed no significant main effect for the refractive group (*F*[2,14] = 0.145, *p* = 0.866), nor interaction between group and other within-subject factors (for all, *F* < 1.391; *p* > 0.25). There were significant main effects for all within-subject factors: quadrant (*F*[3,42] = 6.009, *p* = 0.002), SF (*F*[3,12] = 328.848, *p* < 0.001), and speed (*F*[1,14] = 62.216, *p* < 0.001). We also found significant interactions between quadrant and SF (*F*[9,6] = 39.263, *p* < 0.001), quadrant and speed (*F*[3,12] = 3.961, *p* = 0.036), SF and speed (*F*[3,12] = 15.571, *p* < 0.001), and significant three-way interaction between quadrant, SF and speed (*F*[9,6] = 8.383, *p* = 0.009).

### The Effect of Myopia on the Peripheral Motion Detection Threshold at 27° Eccentricity

**FIGURE 3:**
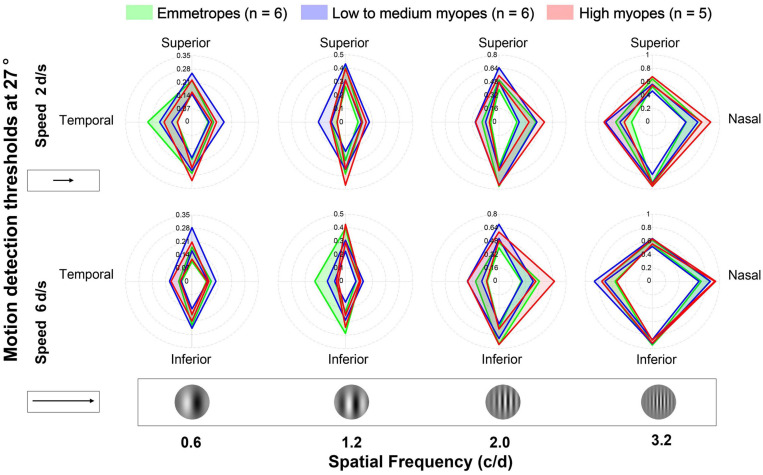
Average motion detection thresholds at 27° for emmetropes, low to medium myopes and high myopes. Data were plotted for different visual fields (in different quadrants), spatial frequencies (in different columns), and speeds (in different rows). The green, blue, and red lines and shadows represent three refractive groups (EM, LM, and HM). The width of the band represents the range between “mean ± SE”.

Figure 3 illustrates the average motion detection thresholds at 27° for the three groups. Overall, no differences were observed among the three groups under any of the conditions in Figure 3. A four-factor mixed ANOVA, with quadrant, SF and speed as within-subject factors and refractive group as between-subject factor, also revealed no significant difference between group (*F*[2,14] = 0.475, *p* = 0.632), nor interaction between group and factors (for all, *F* < 0.403; *p* > 0.097). There were significant main effects for quadrant (*F*[3,42] = 19.139, *p* < 0.001) and SF (*F*[3,12] = 233.346, *p* < 0.001), and significant interactions between quadrant and SF (*F*[9,6] = 26.16, *p* < 0.001), quadrant and speed (*F*[3,12] = 7.388, *p* < 0.001), SF and speed (*F*[3,12] = 19.394, *p* < 0.001) and three-way interaction between quadrant, SF and speed (*F*[9,6] = 2.023, *p* = 0.042). While no significant main effect for speed (*F*[1,14] = 1.26, *p* = 0.281) was found.

### Correlation Analysis of the Motion Detection Threshold and the Spherical Equivalent of Myopes

**FIGURE 4:**
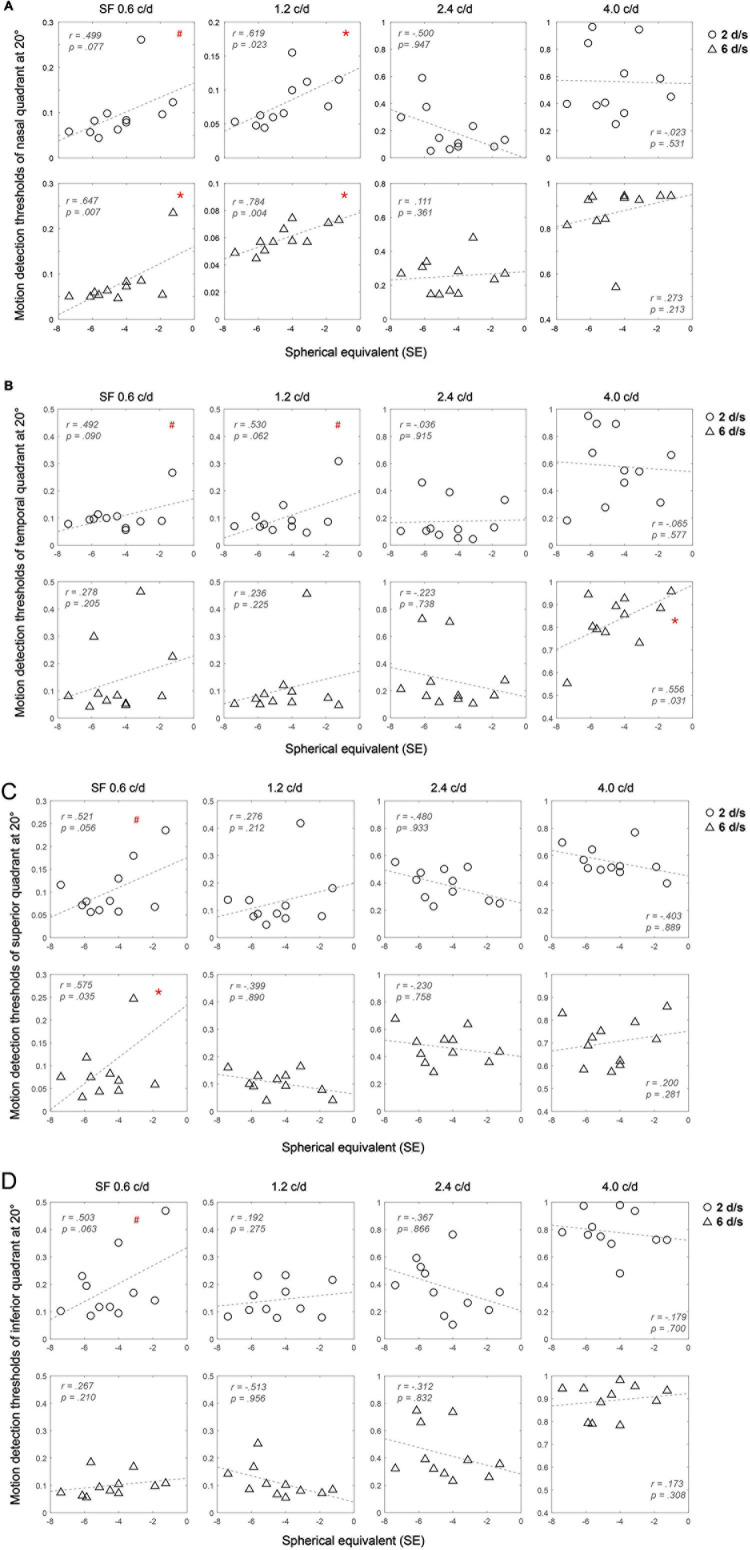
Relationship between the motion detection thresholds and the spherical equivalent (SE) of myopes in the nasal **(A)**, temporal **(B)**, superior **(C)** and inferior **(D)** visual field at 20°. Each point represents one participant, the circles represent 2 d/s, and the triangles represent 6 d/s. Pearson correlation coefficients and empirical *p* values from permutation tests (based on 10,000-time simulation) are shown. #*p* < 0.1; *p < 0.05.

We further performed Pearson correlation analysis and used empirical *p* values from permutation tests to determine the significance of the correlation between the motion detection threshold and the spherical equivalent of myopic subjects in LM and HM. Results at 20° are shown in Figure 4. At 20°, significantly positive correlations between motion detection thresholds and refractive error were found at low SF (i.e., 0.6 and 1.2 c/d) and mainly high speed (6 d/s) in the nasal visual field (for all, *p* < 0.05). This means that patients with higher myopia had lower motion detection thresholds. Such pattern was also found at low SF (0.6 c/d) and high speed (6 d/s) in the superior visual field (*r* = 0.575, *p* = 0.034). While at high SF (4.0 c/d), significant correlation only occurred at high speed (6 d/s) in the temporal visual field (*r* = 0.556, *p* = 0.032). At 27°, no significant correlation was found in any condition (for all, *p* > 0.1). Results for 27° are attached in Supplementary Material.

## Discussion

In this study, we investigated the effect of myopia on peripheral motion detection in young adults, and to see if it varied with eccentricity, spatial frequency, speed, as well as location in the visual field. We found no statistically significant difference in the peripheral motion detection threshold between emmetropes, low to medium myopes, and high myopes. Further analysis revealed that lower motion detection thresholds were associated with higher myopia, mostly for low spatial frequency target, at 20° in myopic viewers.

Although there was evidence that peripheral visual deficit existed in myopes compared to emmetropes, it was contrast-dependent. For example, Ehsaei et al. (2013) found reduced peripheral acuity at a high contrast level (100%) but not at a low contrast of 14%. Contrast detection thresholds in the periphery were found not to differ between myopes and emmetropes in Kerber et al. (2016) study where they measured peripheral contrast detection thresholds binocularly by using vertical Gabor stimuli presented at three eccentricities, 8°, 17°, and 30°. These findings, together with ours, suggest that the difference of peripheral perception between myopes and emmetropes is minimal at low contrast.

The fact that no statistical difference in the behavioral task performance between myopes and emmetropes cannot rule out the potential effect of myopia on peripheral motion perception. In fact, we still found significant correlations between the spherical equivalent of myopia and the peripheral motion detection threshold, mostly in the nasal and superior visual fields at low SF. The correlations suggest the myopic impact on peripheral motion perception. This finding is consistent with a previous study in which Kuo et al. (2018) used random-dot patterns to assess dot motion perception using Dmin, Dmax, and motion coherence tasks in both central and peripheral visual fields in young myopic and emmetropic adults. They also found that the Dmin threshold in the superior-temporal visual field was correlated positively with the axial length and negatively with the macular thickness of the corresponding retina, despite the fact that no difference was found between myopes and emmetropes, regardless of the tasks used, in the periphery. This correlation suggested that the severer myopia, the worse the performance of the Dmin task at the near periphery. Although the direction of correlations between the severity of myopia and the peripheral motion detection task performance was opposite to that in our study, it could be due to, first, the difference in eccentricity which was 3.65° in their study, 20° and 27° in our study; second, the nature of the task which was global motion processing in their study and local motion processing in ours; and third, the SF of the stimuli that covered the full range by the dots in their study and more narrowly filtered in our Gabor stimuli for the current study, whereas the myopic impact on contrast sensitivity has been demonstrated to be uneven across the spatial frequency range (Risse et al., 1996; Jaworski et al., 2006).

The negative correlations we found between the myopia severity and the peripheral motion detection threshold mostly at the low spatial frequency in the nasal and superior visual field have some interesting implications about myopic vision and the myopization process. First, findings from visual search tasks suggested that myopes tend to adopt the local processing strategy over the global processing strategy (Mascetti et al., 2001), or focus their attention more locally (McKone et al., 2008; Kerber et al., 2016) compared to their emmetropic counterparts. Therefore, patients with severer myopia may perform better in the current peripheral motion detection task which required local instead of global processing. Second, myopic vision may try to compensate for its loss in the central vision, especially at high SF (Risse et al., 1996; Jaworski et al., 2006), by improving peripheral vision at low SF. Compensation of central vision loss by peripheral vision has been discovered in several brain plasticity studies (Cummings et al., 1985; Timberlake et al., 1986; Maniglia et al., 2020). Third, there is consensus that visual performance varies in different parts of the visual field (Wertheim, 1980; Fahle and Schmid, 1988). For example, it was found that peripheral visual acuity was better in the nasal and superior retinal regions than the temporal and inferior regions for both myopic and emmetropic groups (Ehsaei et al., 2013). The finding of the current study that correlations between myopia severity and the peripheral motion detection were mostly found in the nasal and superior visual field and only at 20°, not 27°, further suggested that the myopic influence on visual performance also varies across the whole visual field. This uneven distribution of the myopic impact in the visual field is also consistent with the finding in Kuo et al. (2018) study that only in the superior-temporal visual field the correlations between Dmin task performance and myopia severity were significant. Besides, the correlation we found was mainly at low SF. This indicated that the effect of myopia varies across the SF range, which is consistent with Diez et al. (2020) research. They found that the changes of accommodation response were SF-dependent, too. In particular, the changes of accommodation response of emmetropes and myopes were similar after the induction of stimulation of high SF, while there was an opposite direction of accommodation response changes between the two groups after low SF stimulation.

In this study, we did not measure or correct the subjects’ peripheral refractive status like some previous studies, which used an adaptive optics vision system (Ghosh et al., 2016; Zheleznyak et al., 2016). Peripheral vision was affected by multiple ocular and physical factors, including refractive error, diffraction, scattering, aberration, and the form of the palpebral fissure (Rosenholtz, 2016). Many studies have shown that refractive errors distribute unevenly across the whole retina, and conventional correction methods including single vision spectacle lenses and soft contact lenses do not correct the refractive errors in the whole visual field evenly either (Shen et al., 2010). It is possible that uncorrected peripheral refraction might have influenced the threshold of the peripheral motion detection. However, previous studies have found that the relative peripheral refraction is larger when the central corrective power used is higher with both contact lenses (Shen et al., 2010; Moore et al., 2017) and spectacles (Lin et al., 2010). This means that more severe myopic eyes corrected with soft contact lenses suffer from more peripheral refractive errors, thus more blur in the peripheral vision. This is opposite to the negative correlation between myopia severity and peripheral motion detection threshold. Thus, the correlation we found could not be completely explained by refractive status in the peripheral retina.

Compared with previous studies, we did not take additional measures [e.g., blind spot (Leibowitz et al., 1972; Johnson and Leibowitz, 1974; Kuo et al., 2018), bright-colored afterimage (McKee and Nakayama, 1984), and pupil tracking camera (Venkataraman et al., 2015), etc.] to monitor subjects’ eye movements. In our experiment, stimulus was randomly presented in one of four visual quadrants to minimize the effect of expected eye movements. Previous studies have shown that the response time of the human eye to the moving target is 150–250 ms on the horizontal meridian, which increased with the decrease of motion speed (Westheimer, 1954), and the latency of a voluntary saccade in a visual search was found to be 200–250 ms (Araujo et al., 2001). This means that the duration of the stimulus-600 ms, was not long enough for the subjects to scan all four possible locations in the four visual quadrants to look for the target with central vision. It was possible that the gaze moved toward the target location unintentionally upon the detection of the target. It was the consequent reaction following the detection of the target with peripheral vision, instead of planned voluntary eye movements to see the target with central vision. In addition, the statistical difference of motion detection thresholds between the two eccentricities, and among the four visual quadrants also indicated that the detection was not performed by central vision.

The viewing distance was 27 cm in our study. We have adjusted eccentricity with viewing distance due to the size of the screen and proximal accommodation (PA). The distance of PA in emmetropes is generally 25 cm, and shorter in myopic patients (Maiello et al., 2014). In order to measure the largest eccentricity and to avoid the occurrence of PA with our experimental setup, 27° were selected as the largest eccentricity to test, and 27 cm as the viewing distance.

The orientations and motion directions of the Gabor targets in the current study were designed intentionally. Previous studies (Berkley et al., 1975; Venkataraman et al., 2016; Zheleznyak et al., 2016) have shown that peripheral vision measured with gratings oriented parallel to the meridian performs better than those using perpendicular gratings. While in the current study, by using Gabors oriented perpendicular to the meridians, we kept the motion direction at each location consistent with the major relative motion direction of environmental objects during locomotion. To keep the location of the target constant, phase-shifting Gabors were used. If the orientation was parallel to the motion direction, one would not tell that the target was moving. Thus, the orientation of the Gabor at each location had to be perpendicular to its motion direction. What’s more, aligning the motion direction with the meridian also avoids bias toward one of the quadrants at the other meridian. For example, a Gabor moving horizontally at the superior location presents a bias toward the temporal or nasal field depending on the viewing eye. To achieve these aforementioned goals, also considering that the orientation sensitivity of different meridian is different (Berkley et al., 1975; Venkataraman et al., 2016; Zheleznyak et al., 2016), we intentionally used Gabors oriented perpendicular to its motion direction.

Note that all our myopic subjects were young adults with stable refraction status. The peripheral motion perception in myopic children whose refractive status is still developing is an interesting topic for further research for two main reasons. First, peripheral defocus plays an important role in myopia progression (Smith et al., 2005, Smith et al., 2010; Mutti et al., 2007). Second, previous studies showed different development rates of peripheral vision from central vision (Bjerre et al., 2014), and more constricted visual field in children compared to adults (Aspinall, 1976). Whether the maturation of peripheral visual functions is affected by myopia and how it is different from the central vision will provide insight into the myopization process.

## Conclusion

In summary, no significant difference was found in the peripheral motion detection threshold between myopic and emmetropic observers, however, we showed significant correlations between the spherical equivalent of myopia and the peripheral motion detection threshold, mostly in the nasal and superior visual fields at low SF, and in the temporal quadrant at high spatial frequency and high speed in myopic viewers at 20°. The higher the myopia, the lower the motion detection thresholds. We speculate it might be related to adaptation and compensation in the process of myopia development. Future research on the effect of myopia on peripheral motion perception in children will contribute to further understanding of the myopization process.

## Data Availability Statement

The raw data supporting the conclusions of this article will be made available by the authors, without undue reservation.

## Ethics Statement

The studies involving human participants were reviewed and approved by The Ethics Committee of the Affiliated Eye Hospital of Wenzhou Medical University. The participants provided their written informed consent to participate in this study.

## Author Contributions

JW, DK, AY, BD, JB, JZ, YG, and ZH conceived the experiments. JW, DK, XY, LW, and YX performed the experiments. JW, DK, ZH, YG, and JZ analyzed and interpreted the data and wrote the manuscript. All authors contributed to manuscript revision, read, and approved the submitted version.

## Conflict of Interest

YG, AY, and BD were employees of Essilor International, Singapore. JB was an Associate Director of Wenzhou Medical University-Essilor International Research Center. The remaining authors declare that the research was conducted in the absence of any commercial or financial relationships that could be construed as a potential conflict of interest.

## Supplementary Material

Correlation Analysis of the Motion Detection Threshold and the Spherical Equivalent (SE) of Myopes at 27°

We also performed Pearson correlation analysis and used empirical *p* values from permutation tests to determine the significance of the correlation between the motion detection threshold and the SE of myopic subjects in LM and HM at 27°. As shown in Supplementary Figure, no significant correlation was found under all conditions (for all, *p* > 0.1).

Supplementary Figure Relationship between the motion detection thresholds and the spherical equivalent (SE) of myopes in the nasal **(A)**, temporal **(B)**, superior **(C)** and inferior **(D)** visual field at 27°. Each point represents one participant, the circles represent 2 d/s, and the triangles represent 6 d/s. Pearson correlation coefficients and empirical *p* values from permutation tests (based on 10,000-time simulation) are shown.
