# Supplementary figures and images for: Is Peripheral Motion Detection Affected by Myopia?
Source: Front Neurosci. 2021 Jun 7;15:683153. doi: 10.3389/fnins.2021.683153 (PMC8215660; doi:10.3389/fnins.2021.683153)

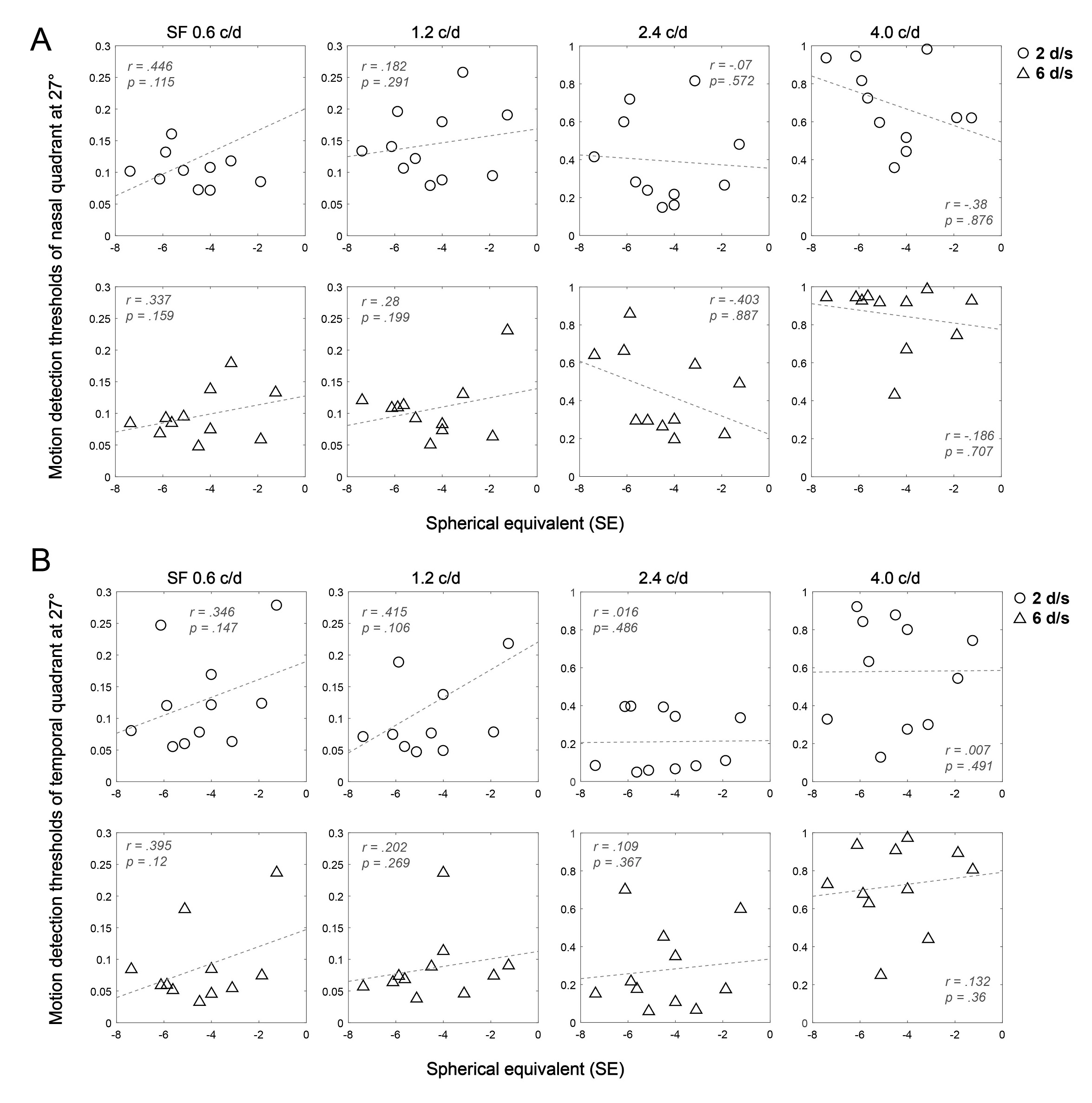

Supplement: Supplementary file 1 [file Image_1.JPEG]

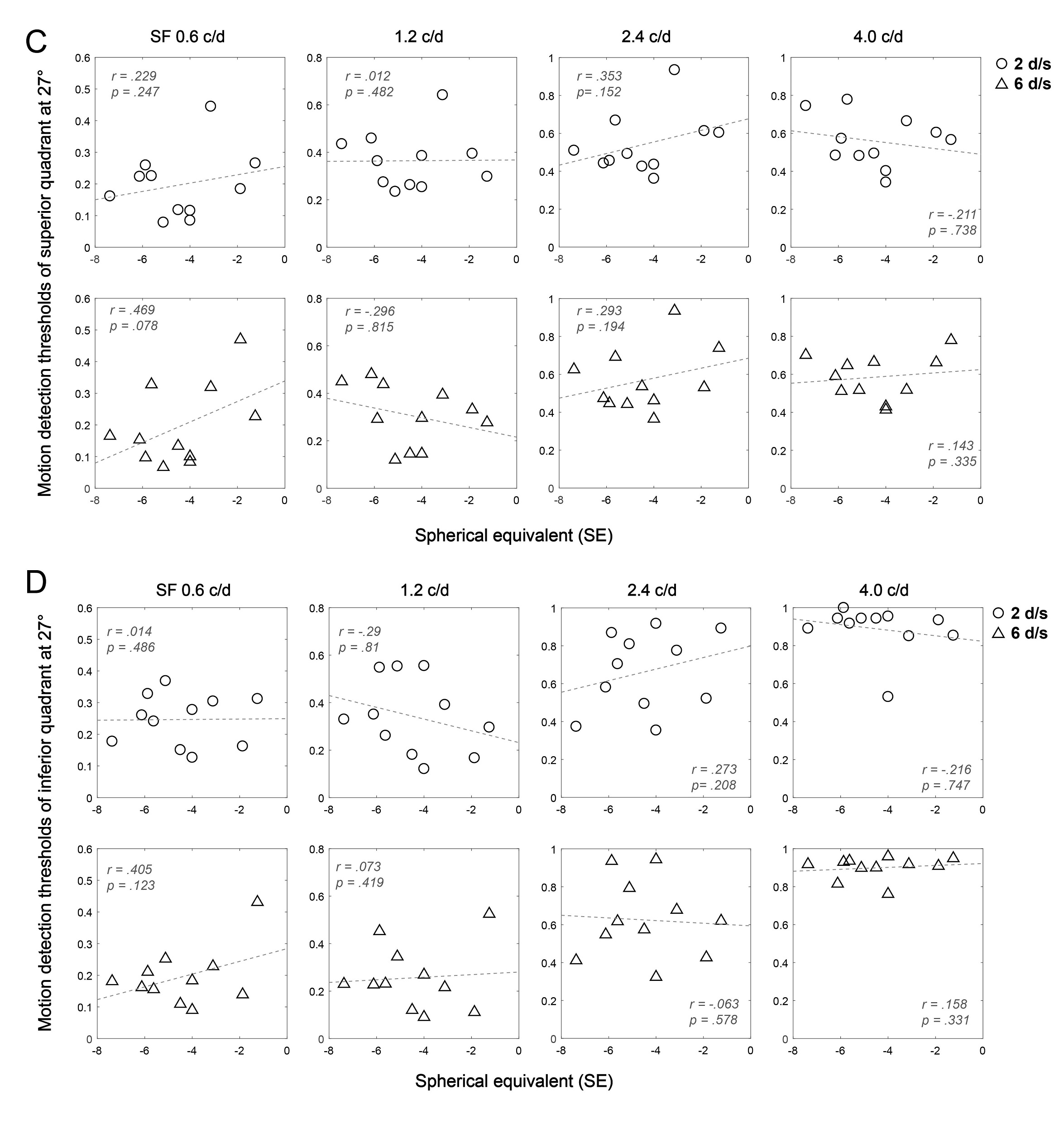

Supplement: Supplementary Figure — Relationship between the motion detection thresholds and the spherical equivalent (SE) of myopes in the nasal (A), temporal (B), superior (C) and inferior (D) visual field at 27°. Each point represents one participant, the circles represent 2 d/s, and the triangles represent 6 d/s. Pearson correlation coefficients and empirical p values from permutation tests (based on 10,000-time simulation) are shown. [file Image_2.JPEG]
